# An assessment of recently published gene expression data analyses: reporting experimental design and statistical factors

**DOI:** 10.1186/1472-6947-6-27

**Published:** 2006-06-21

**Authors:** Peyman Jafari, Francisco Azuaje

**Affiliations:** 1 Department of Biostatistics and Epidemiology, Faculty of Health, Shiraz University of Medical Sciences, Shiraz, Iran; 2 School of Computing and Mathematics and Computer Science Research Institute, University of Ulster, BT37 0QB, UK

## Abstract

**Background:**

The analysis of large-scale gene expression data is a fundamental approach to functional genomics and the identification of potential drug targets. Results derived from such studies cannot be trusted unless they are adequately designed and reported. The purpose of this study is to assess current practices on the reporting of experimental design and statistical analyses in gene expression-based studies.

**Methods:**

We reviewed hundreds of MEDLINE-indexed papers involving gene expression data analysis, which were published between 2003 and 2005. These papers were examined on the basis of their reporting of several factors, such as sample size, statistical power and software availability.

**Results:**

Among the examined papers, we concentrated on 293 papers consisting of applications and new methodologies. These papers did not report approaches to sample size and statistical power estimation. Explicit statements on data transformation and descriptions of the normalisation techniques applied prior to data analyses (e.g. classification) were not reported in 57 (37.5%) and 104 (68.4%) of the methodology papers respectively. With regard to papers presenting biomedical-relevant applications, 41(29.1 %) of these papers did not report on data normalisation and 83 (58.9%) did not describe the normalisation technique applied. Clustering-based analysis, the *t*-test and ANOVA represent the most widely applied techniques in microarray data analysis. But remarkably, only 5 (3.5%) of the application papers included statements or references to assumption about variance homogeneity for the application of the *t*-test and ANOVA. There is still a need to promote the reporting of software packages applied or their availability.

**Conclusion:**

Recently-published gene expression data analysis studies may lack key information required for properly assessing their design quality and potential impact. There is a need for more rigorous reporting of important experimental factors such as statistical power and sample size, as well as the correct description and justification of statistical methods applied. This paper highlights the importance of defining a minimum set of information required for reporting on statistical design and analysis of expression data. By improving practices of statistical analysis reporting, the scientific community can facilitate quality assurance and peer-review processes, as well as the reproducibility of results.

## Background

The analysis of large-scale gene expression has become a fundamental approach to functional genomics, the identification of clinical diagnostic factors and potential drug targets. DNA microarray technologies provide exciting opportunities for analysing the expression levels of thousands of genes simultaneously [1]. A fundamental objective in microarray data analysis is to identify a subset of genes that are differentially expressed between different samples (e.g. conditions, treatments or experimental perturbations) of interest. However, despite the exponential growth of these studies published in journals, relatively little attention has been paid to the task of reporting important experimental design and analysis factors. Nowadays, researchers, clinicians and decision makers rely on such publications, an implicitly on the peer review process, to assess the potential impact of research, reproduce findings and further develop the research area. Information on experimental design and the correct use of statistical methods is fundamental to aid the community in correctly accomplishing their interpretations and assessments.

Over the past few decades the medical research disciplines, especially the area of clinical trials, have widely emphasised the importance of rigorous experimental design, statistical analysis implementation and the correct use of statistics in peer-reviewed publications [2-6]. Although the general understanding of basic statistical methods (e.g. *t*-test, ANOVA) has improved in these disciplines, some errors regarding their sound application and reporting can still be found. For instance, the *t*-test and ANOVA are fairly robust to moderate departures from its underlying assumptions of normally-distributed data and equality of variance (homogeneity) except in the presence of very small or unequal sample sizes, which can considerably decrease the statistical power of the analyses [7-10]. In order to promote a more rigorous application and reporting of data analyses in the area of clinical trials, the *Consolidated Standards of Reporting Trials *(CONSORT) have been adopted. CONSORT has significantly assisted researchers in improving the design, analysis and reporting of clinical trials [11]. This is an example of how a community-driven effort can help to improve the reporting of scientific information. Moreover, this instrument has shown to be helpful to authors, reviewers, editors and publishers to improve the readers' confidence in the scientific quality, relevance and validity of the studies published. We and others argue [12,13] that there is still a need for more rigorous approaches to reporting information relevant to gene expression data analysis. Therefore, it is important to have a closer look at the level achieved by recently published papers in connection to fundamental factors for correctly justifying, describing and interpreting data analysis techniques and results.

The main objective of this investigation is to assess the reporting of experimental design and statistical methodologies in recently published microarray data analysis studies. Among the experimental design factors under study are sample size estimation, statistical power and normalisation. This paper also provides insights into the design of studies based on well-known statistical approaches, such as *t*-test and ANOVA. Our research also examined how papers present fundamental statistical justifications or assumptions for the correct application of the *t*-test and ANOVA, which are widely applied to gene expression data analysis.

## Methods

*PubMed *[14] was used to identify papers presenting results on gene expression data analysis between 2003 and 2005 using "gene expression data" as the query expression. A manual selection process was implemented in which the following categories of papers were excluded: a) review articles; b) commentaries and brief communications; and c) editorial notes including correspondence to editors. Furthermore, we excluded papers concentrating on: a) Web servers, b) databases, and c) software tools. Full papers were then obtained from different journals [see Additional file 1]. The reporting of the following factors was examined: a) type of study (two main types: papers focused on the presentation of new analysis *methodologies *and biomedical-relevant *applications*); b) reporting of methods of sample size calculation and statistical power; c) reporting of data standardisation (i.e. normalisation) and method of normalisation applied; d) description of data analysis techniques applied; e) discussion about missing values; f) explicit statement of directionality (i.e. one-sided or two-sided test); g) explicit statement of hypothesis and alternative; and h) reference to software tools applied for implementing data analyses. In this study application papers refer to any paper whose main contribution is the generation or testing of biological or biomedical hypotheses, including potential diagnostic, prognostic and therapy design applications, as well as biologically-relevant discoveries. Methodology articles emphasize the presentation of a novel, problem-specific (experimental or computational) method or procedure, which may drive a biologically-relevant advancement or discovery.

In connection to the description of data analysis techniques applied, we concentrated on the assessment of techniques or models that were fundamental to obtain key findings in the application and methodology papers. With regard to the discussion of missing data estimation methods, we targeted the application of previously-published imputation or estimation methods.

We examined the two main categories of papers on the basis of the factors defined above. For all the factors, except for factor d), we asked whether or not a factor was reported in each paper. In relation to factor d), we reviewed the techniques applied and then organised the papers into groups according to major data analysis paradigms or approaches. Table 1 describes the factors assessed along with key references, which may provide the reader with further details about these concepts and relevant approaches.

**Table 1:** Definition of factors assessed in gene expression data analysis papers.

**Factor**	**Brief definition or question of interest**	Key references*
Sample size	Estimation of the number of arrays required in order to identify significantly, differentially expressed genes.	[15–26]
Statistical power	Ability of a study to detect a true difference between genes, biological category or condition	[2,24,27–28]
Normalisation	Does the paper report normalisation of data? (yes or no)	[29–32]
Normalisation method	Does the paper describe how sources of variation were removed or data standardisation method, e.g. total intensity normalisation, normalisation using regression techniques, normalisation using ratio statistics etc.	[29–32]
Test directionality	Explicit statement of directionality of the statistical test applied, i.e. one-sided or two-sided test	[33–35]
Hypothesis and alternative	Explicit statement of null (H_0_) or alternative hypothesis (H_1_)	[36–39]
Missing values	Report of missing values, report of estimation of missing values or description of method for estimating missing values.	[40–42]
Software	Which software, programs or tools were used for statistical analysis?	[43–44]
Analysis technique	Which statistical approaches were used for gene expression data analysis?	[1,45–47]
Homogeneity of variances	Does the paper report the equality of variances assumption for the application of ANOVA and *t*-test?	[48–49]

* Review articles that may be useful to introduce the reader to these concepts and relevant approaches.

## Results

We reviewed papers published in Medline-indexed journals. Among these papers 152 (51.9%) concentrated on the presentation of new methodologies for gene expression data analysis, and 141 (48.1%) papers mainly contributed application studies, e.g. discoveries directly relevant to molecular biology and clinical studies. The definition of these paper categories was provided above.

Our results show that none of the 293 applications and methodology papers reported approaches to sample size calculation. Moreover, none of these papers reported information relating to the statistical power of their analyses. Only 23 (7.8%) of the papers (9 application and 14 methodology papers) presented discussions about the limitations of small sample sizes in their analyses of real data. Among the methodology papers, only 9 (5.9%) manuscripts provided evidence that their analyses techniques were adequate (e.g. exhibiting good statistical power) for small sample sizes. Only 1 of the application papers discussed statistical power and sample size factors. Among the methodology papers, 94 (61.8%) used real data for assessing the data analysis methodologies or techniques proposed. Three of the methodology papers (2%) only used simulated data to support their evaluations; and 55 (36.2%) papers analysed both real and simulated data for evaluating the methodologies proposed. Table 2 shows the reporting of normalisation and description of normalisation techniques for methodology and application papers. It indicates a lack of information on normalisation procedures applied.

**Table 2:** Reporting normalisation and techniques implemented in published methodology and application papers

Methodology papers				Application papers			
Reporting normalisation		Description of method of normalisation		Reporting normalisation		Description of method of normalisation	

Yes (%)	No (%)	Yes (%)	No (%)	Yes (%)	No (%)	Yes (%)	No (%)
95 (62.5)	57 (37.5)	48 (31.6)	104(68.4)	100(70.9)	41(29.1)	58(41.1)	83(58.9)

Among the 141 application papers, 11 papers (7.8%) did not report the statistical methods used in their data analyses. Clustering-based analysis, the *t*-test and ANOVA represent the most widely applied techniques in microarray data analysis studies (Table 3). Table 3 also shows that from the 141 application papers, 68 papers applied statistical analyses based on the *t*-test (21 papers) or ANOVA (47 papers). However, our review showed that only 5 (3.5%) of the application papers discussed variance homogeneity assumptions in their analyses. Moreover, only 7 (4.6%) of the methodology papers presented statistical justifications for the application of either ANOVA or the *t*-test.

**Table 3:** Main types of statistical methods applied in microarray data analysis studies.

Technique	Application papers (%)*	Methodology papers (%)
*t*-test	21 (14.89)	11 (7.24)
ANOVA	47 (33.33)	22 (14.47)
Data clustering	56 (39.72)	75 (49.34)
Supervised classification	5 (3.55)	37 (24.34)
Mixed classification models	3 (2.13)	12 (7.89)
Nonparametric tests	11 (7.80)	6 (3.95)
Regression analysis	7 (4.96)	11 (7.24)
Correlation-based analyses	23 (16.31)	4 (2.63)
Fuzzy logic methods	0 (0.00)	4 (2.63)
Fisher-exact tests	5 (3.55)	5 (3.29)
PCA	7 (7.96)	4 (2.63)
Discriminant analysis	4 (2.84)	4 (2.63)
Time series analysis	0 (0.00)	6 (3.95)
Meta analysis	2 (1.42)	1 (0.66)
Other methods	9 (6.63)	22 (14.47)

* Percentages calculated in relation to each paper category separately. For example, in connection to the use of *t*-test in application papers, the table indicates that 21 application papers (out of 141), i.e. 14.89 %, used this technique.

Our results showed that among the methodology and application papers, 133 (87.5%) and 115 (82%) did not report the directionality of the tests (one-sided or two-sided test) respectively. Also among the methodology and application papers, only 19 (12.5%) and 26 (18%) included discussions about missing values (report of missing values, estimation of missing values or description of methods for missing value estimation) respectively. Explicit statements of hypothesis and alternative hypothesis were reported in only 43 (28%) and 29 (20.6%) methodology and application papers respectively. In addition, of the 141 application papers, only 52 (36.9%) included sections or sub-sections to describe data analysis methods applied.

As shown in Table 4, 39 methodology and 46 application papers did not adequately report the software tools used to implement their data analyses. Our review found that among these 85 papers: 53 did not discuss software or algorithms applied to data analysis at all, and 24 papers presented incomplete or unclear descriptions of the software or algorithms applied (i.e. the reader would not be able to identify the type of statistical methodology or software package applied). Only 8 papers from the methodology and application categories offered full software implementations of the statistical analysis algorithms applied upon request from the authors. However, 208 papers included information on software tools or algorithms applied. A closer look at these 208 papers reveals the application of 274 software tools or programs either implemented by the authors or obtained from other resources to perform their data analyses. Table 5 shows the most used software packages, tools or programs. It indicates an increasing tendency to make software tools available on the Web. It also highlights the diversity of standalone and proprietary packages and implementations applied for data analysis.

**Table 4:** Reporting on software tools or programs for data analysis included in Table 3.

Methodological papers		Application papers	
Yes (%)	No (%)	Yes (%)	No (%)
113 (74.3)	39 (25.7)	95 (67.4)	46 (32.6)

**Table 5:** The most applied software tools

**Software systems**	**Usage Frequency**
Web-based implementations*	40
R	31
MATLAB	16
MAS	16
SAS	16
GeneSpring [50]	14
Excel	12
TreeView [51]	12
S-PLUS	9
SPSS	8
Standalone programs implemented in C++ or Java	12
Gene Cluster (Cluster) [51]	10
Significance Analysis of Microarrays (SAM) [52]	6
BioMiner	2
Other proprietary implementations	73

* implemented by authors or originating from related studies

## Discussion

Our assessment suggests that published papers lack relevant information regarding the determination of sample sizes and statistical power in microarray data analysis studies. These studies often involve hundreds or thousands of genes and only a fraction of genes are expected to be differentially expressed. Therefore, genes that do not show clear patterns of differential expression are filtered out, by performing statistical group comparisons. However, if the subjects or arrays (sample size) have not been properly estimated before the statistical comparisons (e.g. ANOVA or *t*-test) then spurious predictions and type II errors (β) can be seriously misleading. In fact, undetected significant differences may be explained by a lack of statistical power for detecting true differences between genes or as a result of inadequate sample sizes (subjects or arrays). Our study showed that very few research studies (i.e. either methodology or application papers) discuss power and sample size requirements in microarray experiments, which are fundamental factors to accomplish the validation of the statistical analyses [15-26].

Our review also shows that although classic ANOVA and the *t*-test are widely applied to the analysis of gene expression data, fundamental statistical assumptions, such as the homogeneity of variances, are seldom mentioned. Therefore, even if we ignore the constraints defined by small sample size in the application of ANOVA and *t*-test, these papers fail to justify their application on the basis of their assumptions of homogeneity of variance. Researchers also have the option of implementing other statistical significance tests that may relax the assumption of homogeneity of variance. Researchers should also be aware of the limitations of the classic *t*-test and ANOVA methods for detecting differential expression patterns, e.g. statistical power and detection of spurious relations. Therefore, relatively more powerful and reliable alternatives may be carefully considered, such as distribution-free tests, linear models with empirical Bayes corrections or other significance analysis techniques for gene expression data.

Furthermore, our results indicate that gene expression data analysis papers should provide additional information on data normalisation methods applied. This important data analysis reporting task deserves more attention in order to support a more accurate interpretation and reproducibility of results. Although previous research [53] has suggested relatively high robustness of microarray data analysis to different types of normalisation techniques, more evidence clearly indicates that prediction outcomes can be significantly affected by the selection of normalisation methods [29-32]. Therefore, we argue that authors should not only indicate that their data have been normalised, but also they should provide details on the normalisation method applied and assumptions.

Our findings show that only 45 (15.4%) methodology and application papers explicitly discussed issues relating missing values, e.g. sources and estimation methods. Gene expression data often contain missing expression values, which may require the application of missing data estimation or imputation techniques to obtain a complete matrix of expression values. Like in the case of data normalisation, authors not only should report on missing values, but also on their approaches to dealing with such a problem. Again this is a crucial factor because different estimation methods may have different effects on the same dataset [40-42,54]. Also our results stress the need to continue encouraging authors to provide adequate descriptions of the software tools or resources applied to implement their data analyses. For instance, 53 (18.1%) of the application and methodology papers examined did not provide any information on the software package or programs used to implement their statistical analyses.

Finally, our review suggests that the above reporting practices may be improved by encouraging authors to provide separate sections or sub-sections focusing on data analysis. Only 36.9% of the application papers, for example, included a section dedicated to these aspects, i.e. detailed discussion of methods, tools, assumptions. A section (or sub-section) on statistical methods should clearly state, for instance, how the sample size was estimated and how the data were analysed in relation to each of the objectives and underlying biological and statistical assumptions made. Such a section should also include information about statistical software or tools applied for data analysis (e.g. origin and availability) and the directionality of the statistical tests applied.

Even when this study did not aim to analyse the possible causes of such relative lack of statistical information reporting standards, it is necessary to stress the importance of ensuring the participation of statisticians in both the design and analysis phases of gene expression studies. However, in some cases this may be accomplished only if adequate provisions and decisions are made during the project formulation and funding assessment phases (i.e. adequate budget considerations should me made to achieve such participation). An interpretation of the results on the reporting of test directionality should also take into account that for many authors it may be common practice not to report test directionality as they may assume that two-sided directionality is the default setting. However, this assumption should not be used to justify the lack of more rigorous reporting practices, which are commonly adopted in other statistics-driven areas, such as medical sciences, epidemiology and clinical trials.

It is also necessary to recognise that the lack of more rigorous reporting standards may be understood in the light of the technical complexities and constraints presented by the area of gene expression data analysis. For example, there is a need for more comprehensive theoretical and empirical studies about the statistical nature of gene expression data in order to help researchers to present deeper discussions on sample size and power analysis. In relation to these factors, one may also argue that, unlike the clinical sciences domain, there is a lack of accepted, comprehensively-validated methods tailored to gene expression data. Therefore, it is fundamental to promote deeper investigations and the generation of robust, user-friendly tools to assist researchers in their approaches to the discussion of these factors.

More investigations on the application and reporting of other important experimental procedures, such as sample pooling prior to hybridization, are required. It has been shown that pooling may significantly affect the quality of data analysis [55]. Our review showed that only 13 (8.6%) methodology and 21 (14.9%) application papers reported pooling procedures in their studies. These figures are in general consistent with previous estimates of the number of datasets catalogued in the *Gene Expression Omnibus Database *using this procedure [56].

Another fundamental analysis factor that continues deserving additional investigations is the application and reporting of P-values adjustments. Our review revealed that only 15 (10.7%) and 28 (18.4%) of the application and methodology papers respectively explicitly reported the P-value adjustment method applied. For instance, among the 141 application papers, 8 (5.7%) and 7 (5%) papers reported the use of Bonferroni and Benjamini-Hochberg adjustment methods respectively. With regard to the methodology papers (152 in total): 14 (9.2%), 12 (7.9%) and 2 (1.3%) papers reported the application of Bonferroni, Benjamini-Hochberg and Hochberg adjustment methods respectively. The selection of a suitable adjustment method depends on the error rate that one wants to control [55]. For example, for controlling family-wise error rates (FWER) Bonferroni and Hochberg are recommended, but for controlling false discovery rates (FDR) Benjamini-Hochberg may be a more appropriate choice [55,57-59].

Our study may be complemented by other reviews on the correct application of evaluation strategies, such as data sampling and significance interpretation [60]. Additional studies may be useful to assess more specific data analysis components, such as cross-validation techniques for estimating predictive performance of supervised classification models in medical diagnosis and prognosis. To further support a deeper understanding on issues relevant to statistical information reporting, the reader is also referred to [44,45,55], which review some of the most representative approaches to analysing gene expression data in different biomedical applications.

Future work may involve an analysis of potentially interesting, significant time-dependent trends relating to statistical information reporting. This may allow the scientific community to assess emergent practices and patterns of knowledge generation and reporting in gene expression data analysis.

## Conclusion

Medical research disciplines, especially the area of clinical trials, have placed relatively more emphasis on the reporting of experimental design, statistical analysis implementation and the correct use of statistics in peer-reviewed publications [2-6] in comparison to the current state in gene expression data analysis.

The present survey indicates that the quality and coverage of information regarding experimental design and statistical analysis in gene expression data-driven studies deserve to be improved. The reporting of statistical power, sample size, normalisation and missing data estimation techniques requires a more rigorous treatment. Poor or incomplete reports may significantly affect our capacity to interpret results and assess the relevance and validity of research studies. Moreover, inadequate reporting of statistical analysis information may increase the likelihood of publishing spurious associations or predictions. By paying more attention to these factors authors will be facilitating quality assurance and peer-review processes, as well as the reproducibility of results, which are fundamental factors for the advancement of scientific and technological development, policy and decision making.

Community-driven efforts such as the MIAME (Minimum Information About a Microrray Experiment) protocol [61] may be useful for motivating or guiding the definition of a well-defined set of requirements for reporting fundamental data analysis and experimental statistical design factors. This research calls for greater discussions involving researchers, editors, publishers and decision makers.

## Competing interests

The author(s) declare that they have no competing interests.

## Authors' contributions

PJ and FA co-designed the study. PJ implemented it by retrieving and reviewing papers and constructing quantitative descriptions of the review. FA selected relevant papers and resources for the analysis. PJ and FA co-wrote the manuscript.

## Pre-publication history

The pre-publication history for this paper can be accessed here:



## Supplementary Material

Additional File 1 **Origin of papers- List of journals** Click here for file
