# Supplementary material for: An assessment of recently published gene expression data analyses: reporting experimental design and statistical factors
Source: BMC Med Inform Decis Mak. 2006 Jun 21;6:27. doi: 10.1186/1472-6947-6-27 (PMC1523197; doi:10.1186/1472-6947-6-27)
Supplement: Additional File 1 — Origin of papers- List of journals [file 1472-6947-6-27-S1.pdf]

Origin of papers examined – List of journals

Am J Hum Genet
Am J Obstet Gynecol
Am J Pathol
Am J Physiol Endocrinol Metab
Appl Microbiol Biotechnol
Artif Intell Med
Biochem Pharmacol
Biochim Biophys Acta
Bioinformatics
Biol Reprod
Biosci Biotechnol Biochem
Biosystems
Biotechnol Bioeng
Biotechnol Lett
Blood
BMC Bioinformatics
BMC Cancer
BMC Genomics
BMC Womens Health
Br J Haematol
Breast Cancer Res
Calcif Tissue Int
Cancer
Cancer Lett
Cancer Res
Carcinogenesis
Cell Microbiol
Chem Res Toxicol
Clin Cancer Res
Comput Biol Chem
Curr Opin Biotechnol
Cytogenet Genome Res
Dev Biol
Dev Dyn
Dev Genes Evol
Development
Diabetes
Endocr Relat Cancer
Environ Health Perspect
Epilepsy Res
Exp Toxicol Pathol
FEBS Lett
Funct Integr Genomics
Genetics.
Genome
Genome Biol
Genome Res
Genomics
Hematology
IEEE Trans Inf Technol Biomed
Immunogenetics
Infect Immun
Int Arch Allergy Immunol
Int Immunopharmacol
Int J Cancer

Int J Med Inform
J Anim Sci
J Appl Physiol
J Bacteriol
J Bioinform Comput Biol
J Biol Chem
J Biomed Biotechnol
J Biomed Inform
J Biopharm Stat
J Chem Inf Comput Sci
J Clin Invest
J Comput Biol
J Exp Med
J Hepatol
J Med Genet J Mol Diagn
J Microbiol Methods
J Mol Med
J Nutr
J Pharmacol Exp Ther
J Theor Biol
J Virol
Kidney Int
Lancet
Leukemia
Medinfo
Mol Biochem Parasitol
Mol Cancer Ther
Mol Psychiatry
Mol Reprod Dev
Mutat Res
Nat Biotechnol
Nat Genet
Nature
Nephrol Dial Transplant
Neural Comput
Neural Netw
Neurobiol Dis
Nucleic Acids Res
Oncogene
Pediatrics
Pharm Res
Physiol Genomics
Plant J
Plant Physiol
PLoS Biol
Proc Natl Acad Sci U S A
Prostate
Reprod Biol Endocrinol
Reprod Toxicol
Semin Cancer Biol
Stat Med
Stem Cells
Toxicol Appl Pharmacol
Toxicol Lett
Toxicol Pathol
Toxicol Sci
Virology
